# Evaluating Symptoms to Improve Quality of Life in Patients with Chronic Stable Angina

**DOI:** 10.1155/2013/504915

**Published:** 2013-12-23

**Authors:** Jeffrey W. Young, Sheila Melander

**Affiliations:** UTHSC College of Nursing, 920 Madison Avenue, Memphis, TN 38163, USA

## Abstract

Chronic stable angina (CSA) is a significant problem in the United States that can negatively impact patient quality of life (QoL). An accurate assessment of the severity of a patient's angina, the impact on their functional status, and their risk of cardiovascular complications is key to successful treatment of CSA. Active communication between the patient and their healthcare provider is necessary to ensure that patients receive optimal therapy. Healthcare providers should be aware of atypical symptoms of CSA in their patients, as patients may continue to suffer from angina despite the availability of multiple therapies. Patient questionnaires and symptom checklists can help patients communicate proactively with their healthcare providers. This paper discusses the prevalence of CSA, its impact on QoL, and the tools that healthcare providers can use to assess the severity of their patients' angina and the impact on QoL.

## 1. Introduction

Chronic stable angina (CSA) is a significant and prevalent problem in the United States that can negatively impact quality of life (QoL). Data from the Centers for Disease Control and Prevention indicate that in 2011 approximately 7.8 million people in the United States aged greater than 20 years experienced angina. More than 500,000 people aged greater than 45 years are diagnosed with CSA each year [1, 2]. In a recent study of CSA incidence, 29% of patients with CSA attending primary care practices experienced at least 1 episode of angina per week [3].

Anginal symptoms can be typical, often described as a burning sensation, pain, pressure, squeezing, or tightness, or atypical, which can include fatigue, indigestion, lightheadedness, nausea, dyspnea, and weakness in addition to pain [4]. Moreover, angina can vary from patient to patient and across the sexes and is present in different parts of the body, including the chest, jaw, neck, shoulder, back, and arms. Atypical symptoms can occur with either gender but are more common in females than males [5–7].

The current guidelines from the American College of Cardiology Foundation and American Heart Association (ACCF/AHA), published in November 2012, define the goals of successful treatment in patients with stable heart disease as maximizing health and function and minimizing the likelihood of death. Specific treatment objectives include maintaining and restoring a level of physical activity, functional capacity, and QoL that is satisfactory to the patient and the complete or nearly complete elimination of ischemic symptoms [4]. Moreover, to achieve these objectives, the ACCF/AHA guidelines highlight the importance of educating patients about the etiology, clinical manifestations, treatment options, and prognosis of their disease, supporting active patient participation in treatment decisions, and using evidence-based pharmacological treatments that improve the patients' health status and survival with minimal side effects. Pharmacological treatment options for CSA include beta-blockers, calcium channel blockers (CCBs), short- and long-acting nitrates, and the late sodium current inhibitor ranolazine. Patients may also undergo revascularization if medically indicated [4, 8].

The first step towards a successful treatment outcome for the patient with CSA is an effective evaluation of anginal severity and its impact on patient functional status and QoL [4]. The purpose of this paper is to raise awareness of the prevalence of CSA, its impact on QoL, and the need for taking an accurate and thorough patient history when assessing patients with probable CSA. In addition, this paper discusses tools that healthcare providers can use to assess the severity of their patients' angina and the impact on QoL, with the aim of providing successful treatment that maximizes survival, eliminates symptoms, and returns the patient to normal functional capacity.

## 2. Chronic Stable Angina Can Negatively Impact Quality of Life

Angina is classified by its impact on physical activity. The four-tiered Canadian Cardiovascular Society Classification (CCSC) System, which measures the limitations to ordinary activity and the timing of angina occurrence, remains the standard method for grading angina in patients with CSA. The classifications range from Class I, defined as “ordinary physical activity does not cause angina/angina occurs with strenuous, rapid, or prolonged exertion at work or recreation,” to Class IV, defined as an “inability to carry out any physical activity without discomfort/anginal symptoms may be present at rest” (Table 1) [9].

Chronic stable angina can cause a decrease in the patient's activity level and their ability to move and participate in normal daily activities and negatively impact their QoL. In a comparison of physical activity and health-related QoL in patients with and without CSA, patients with angina curtailed leisure-related physical activity and became more sedentary to avoid an anginal episode, which in turn impaired their overall health and perception of QoL (Table 2) [10]. The results of this study found that patients with stable angina have lower health-related QoL in multiple domains (physical function, general health, and vitality), impaired exercise performance, and lower levels of physical activity related to leisure compared with patients without stable angina. In the Coronary Artery Disease in General Practice (CADENCE) Study, which investigated the impact of angina on QoL among patients with stable angina attending a primary care practice, approximately 29% of patients experienced at least 1 episode of angina per week, which was associated with worsened QoL and greater limitations on physical activity compared with patients with minimal angina (<1 episode/week over the preceding 4 weeks) [3]. The results of this study demonstrate the important association between symptoms of angina and the health status of patients. Patients with a history of peripheral artery disease, heart failure, and female sex were more likely to have at least 1 episode of angina per week. These data are important in light of earlier studies showing that a reduction in exercise tolerance is associated with an increase in the risk of mortality in patients with cardiovascular (CV) disease [11, 12]. A comparison of the prognostic value of exercise tolerance in patients with CSA to risk factors of death (pack-years of cigarette smoking, hypertension, history of congestive heart failure, or myocardial infarction) found that peak of exercise capacity was the best predictor of death in both patients with and without CV disease. An increase in exercise tolerance can improve a patient's ability to perform daily functions, with every one metabolic equivalent (MET) increase in exercise capacity correlating to a 12% improvement in survival [11].

In addition to improving exercise tolerance, the ACCF/AHA guidelines recommend that patients with stable heart disease be educated about the importance of lifestyle modifications, such as smoking cessation, BP control, and weight, lipid, and diabetes management in improving their QoL [4]. Increased exercise tolerance may also reduce CV risk and enhance QoL in patients with CSA who have comorbid type 2 diabetes mellitus (T2DM), and exercise is recommended by the American Diabetes Association for improving QoL and blood glucose control and contributing to weight loss in patients with T2DM [13]. In patients with T2DM, a structured exercise program has been shown to lower glycosylated hemoglobin by 0.66% and may reduce the risk of diabetic complications [14].

## 3. Assessing Quality of Life in Patients with Chronic Stable Angina

### 3.1. The Role of Nurse Practitioners and Physician Assistants in Patient Care

An important aspect of assessing QoL in patients with CSA is optimal communication between the patient and their healthcare provider. Patients with CSA can vary in their tolerance for symptoms, making it challenging for healthcare providers to provide an accurate assessment of their condition [15]. For example, in the CADENCE study, a marked discordance was observed between the physician and patient perspective on the effect of angina on QoL. While physicians classified 61% of patients as having minimal impediment in physical activity (CCSC Class I) and considered patients to be optimally controlled in 80% of cases regardless of the frequency of their anginal episodes, only 52% of patients reported no angina and 47% reported no diminished QoL with increased frequency of angina [3].

An added challenge for healthcare providers is patients who do not engage in physical activity in order to avoid anginal episodes. Although these patients may become less symptomatic, their increasingly sedentary lifestyle may promote further declines in physical function and QoL [10]. Patients with chronic conditions tend to “normalize” over time and may not realize the extent to which they have become sedentary. As a result, they are unlikely to proactively communicate this information to their healthcare provider. For example, a female patient who is prescribed a beta-blocker for her CSA may report no episodes of chest pain at her 6-month follow-up with her physician and subsequently would be advised to continue with her medication as prescribed until her next follow-up in 6 months. However, in her daily routine she may participate in behaviors that reduce her symptoms, such as choosing the closest parking spot to the entry of her grocery store because she experiences shortness of breath if she walks long distances. As another example, a male patient who underwent percutaneous coronary intervention for CSA may report no chest pain or shortness of breath at his 1-year follow-up with his physician. However, he allows his neighbor to continue mowing his lawn because of his fatigue and lack of energy. These two cases are great illustrations of how people with CSA downregulate their lives without openly reporting it to healthcare providers. Nurse practitioners (NPs) and physician assistants (PAs) have an important role in evaluating patients' condition, as they are likely to be more involved in patient care and may have more opportunity than physicians to spend time with patients and elicit information regarding their experiences.

### 3.2. Tools to Assess Quality of Life

Successfully treating CSA begins with an accurate assessment of the severity of a patient's angina, the impact on their functional status, and their risk of CV complications [4]. Generic health status questionnaires, such as the Short Form 36, may not focus on symptoms that are specific to coronary artery disease (CAD); therefore, disease-specific tools that measure CAD are important when assessing the health status of patients with CAD [16]. Several tools have been designed to facilitate assessment of patient-reported functional status and QoL of patients with CAD in the research and clinical settings. These include the Seattle Angina Questionnaire (SAQ), the MacNew Heart Disease Health-Related QoL Questionnaire, the Ferrans and Powers QoL Index, the Duke Activity Status Index (DASI), and the Speak From The Heart Chronic Angina Checklist.

The SAQ is a self-administered disease-specific measure for patients with CAD that is demonstrably valid, reproducible, and sensitive to clinical change [16]. It is used extensively in the research trial setting to quantify the symptoms, functional limitations, and QoL of patients with stable heart disease in the previous 4 weeks [16, 17]. Use of the SAQ can help healthcare providers identify patients at high risk for morbidity and mortality and can also help identify those patients who may require more aggressive medical therapy or revascularization [17]. Spertus and colleagues evaluated the prognostic utility of the SAQ among patients with CAD and found that a patient's health status (symptoms, QoL, and physical function) was a strong predictor of 1-year mortality and hospitalization for acute coronary syndrome (ACS) [17]. Increased risk of mortality and ACS hospital admissions were associated with lower SAQ scores.

The MacNew Heart Disease Health-Related QoL Questionnaire, also used in patients with heart disease, evaluates the effect of coronary heart treatments on daily activities and physical, emotional, and social functioning [18, 19]. This self-administered questionnaire generates an overall score from 27 questions that govern physical limitations, emotional and social function, and angina symptoms experienced in the previous 2 weeks, and it has been used in multiple clinical studies in patients with heart disease [20, 21]. Assessing patients' health status during hospitalization for angina can be helpful in predicting those patients who may experience a worsened QoL 6 months later [21]. Heller and colleagues found that, among 303 patients who were hospitalized with a diagnosis of acute myocardial infarction or angina and completed a follow-up disease-specific QoL questionnaire at 6 months, scores were consistently lower in patients with angina [21].

The Ferrans and Powers QoL Index, which can be used in either a self-administered or interview format, measures both the satisfaction with and the importance of various aspects of life [22]. This QoL index produces an overall QoL score and four additional scores in the health and functioning, psychological/spiritual, social and economic, and family domains. This index has been used to evaluate QoL in multiple research trial settings in patients with disorders that include CAD, chronic fatigue, and migraines [22–25]. The QoL Index instrument was used to assess life satisfaction among 47 subjects with CAD who completed the instrument 6 to 8 weeks following a coronary event. A strong relationship between psychosocial functioning and life satisfaction was observed [25].

Another assessment of functional capacity used in the research setting is the DASI. It is self-administered and uses 12 questions to assess self-reported measures of physical capacity to estimate peak METs, gauge the patient's functional capacity, and assess aspects of QoL [26]. The DASI was administered to 50 subjects undergoing exercise testing with measurement of peak oxygen uptake and 50 subjects in a control group. The DASI was found to be a valid measure of functional capacity; there was a significant correlation between the DASI and peak oxygen uptake [26].

**Figure 1:**
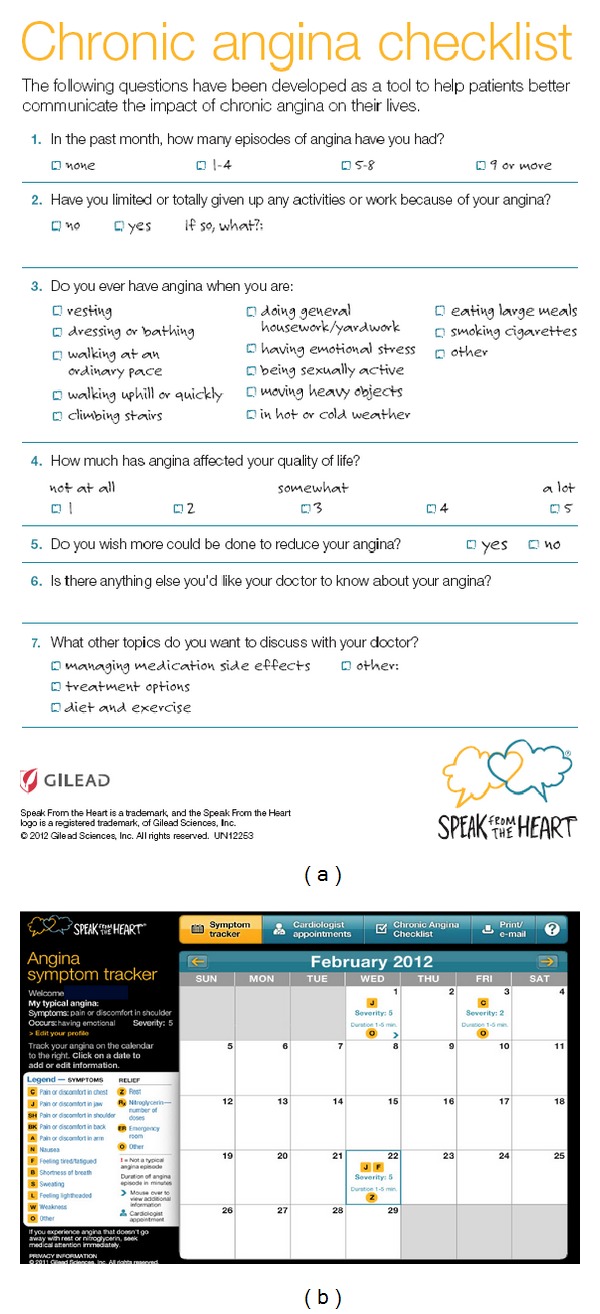
The Speak from the Heart chronic angina checklist and symptom tracker. Available at http://www.speakfromtheheart.com/ (for patients) [27] or at http://www.helpthemspeak.com/ (for healthcare providers).

The Speak From The Heart Chronic Angina Checklist is a self-administered seven-item questionnaire that allows patients to share with their healthcare provider how angina is affecting their QoL, by logging information about each anginal episode (Figure 1(a)) [27]. Designed primarily for use in a clinical setting, this tool also provides the patient with an interactive symptom tracker that allows accurate documentation of the occurrence and frequency of angina episodes (Figure 1(b)). These tools let patients document the frequency of angina, the impact of angina on daily activities, and the impact of angina on QoL and share this information with their healthcare provider [27].

Although many of the tools discussed are used primarily in the research trial setting, the ACCF/AHA guidelines recommend the formal assessment of a patient's disease-specific health status and that these tools be used serially in clinical practice to assess and monitor the effectiveness of antianginal medications, revascularization, and QoL in patients with stable heart disease [4].

## 4. Clinical Insights 

As physicians and patients can have different impressions of the impact of angina on patient QoL [3], healthcare providers need to be aware of additional signs or atypical symptoms in their patients with CSA. Effective communication and clinical assessment of the severity of patient symptoms and functional status are necessary to ensure that patients receive optimal therapy. In addition to the tools mentioned above, questions that go beyond “Are you experiencing any chest pain or shortness of breath?” (Table 3) may be necessary to determine whether a patient is receiving the treatment necessary to attain the ACCF/AHA treatment objectives of the complete or nearly complete elimination of anginal chest pain and restoration or maintenance of a level of activity, functional capacity, and QoL that is satisfactory to the patient [4].

For the NP and PA, asking the appropriate questions and taking an appropriate and thorough history is the key to the assessment of angina. Just because patients are not complaining of “chest pain” does not mean they are not having angina. These patients are at risk of a “missed diagnosis.” The majority of patients with chronic angina do not present with classic chest discomfort but with atypical symptoms that include but are not limited to fatigue, dyspnea, lightheadedness, weakness, nausea, neck pain, shoulder pain, mid- and lower back pain, and arm pain (left or right). These presentations are especially common in women. As angina symptoms typically occur at the end of the ischemic cascade, patients undergo a biochemical alteration and a decrease in relaxation, contraction, and diastolic filling before having symptoms. As such, appropriate identification of symptoms when they do occur is of the utmost importance. Asking the questions outlined in Table 3 as well as using tools that facilitate interaction with the patient in the clinical setting is very important for proper patient assessment.

Moreover, patients are more apt to withhold telling their cardiologist they are having angina for fear of going back to the catheterization laboratory and are much more likely to communicate openly with the NP or PA (if asked) about any symptoms of typical or atypical angina they may be experiencing, as NPs and PAs are more apt to maximize medical therapy before sending patients back to the catheterization laboratory.

## 5. Treatment Options for Chronic Stable Angina

Current pharmacological options for CSA include beta-blockers, CCBs, short- and long-acting nitrates, and ranolazine [4, 8]. Selection of optimal therapy for CSA is critical, as patients have been shown to experience symptoms in the year after being prescribed antianginal therapy [28]. The antianginal efficacy and safety of beta-blockers and CCBs have been demonstrated in several randomized, controlled clinical trials [29–44]. Beta-blockers and CCBs help reduce symptoms of angina, decrease the frequency of anginal episodes, increase exercise duration, prolong the time to ST-segment depression following exercise testing, and improve QoL in patients with CSA [8, 45, 46]. Although these agents are generally well tolerated, some therapies can also have hemodynamic effects; for example, CCBs lower blood pressure while beta-blockers and nondihydropyridine CCBs can decrease heart rate (Table 4) [8, 47, 48]. Therefore, beta-blockers should be avoided or used with caution in patients with hypotension and they are contraindicated in patients with significant sinus bradycardia and partial atrioventricular block [4]. Therapy with beta-blockers can also reduce exercise capacity and impair sexual function in some patients [49, 50]. Similar to beta-blockers, CCBs also exert their effects by lowering blood pressure and heart rate; therefore, nondihydropyridine CCBs are contraindicated in patients with left ventricular systolic dysfunction with or without heart failure and in patients with heart block and disorders of the sinus node [4].

Long-acting nitrates are recommended when initial therapy with beta-blockers or CCBs is contraindicated or poorly tolerated [4]. The antianginal efficacy and safety of long-acting nitrates have been demonstrated in several randomized, controlled clinical trials [51–56]. Long-acting nitrates decrease the frequency of anginal episodes and increase exercise duration in patients with CSA but may be ineffective in improving QoL [57, 58]. Nitrate therapy can also lower blood pressure, with prolonged nitrate therapy leading to the development of tolerance [48]. A minimum 12-hour nitrate-free interval must be maintained every 24 hours to avoid the development of nitrate tolerance. In addition, nitrate therapy is absolutely contraindicated in patients prescribed phosphodiesterase-5 (PDE-5) inhibitors, as the concomitant use of nitrates and PDE-5 inhibitors can potentiate hypotension [4, 57].

Ranolazine, a sodium channel inhibitor, is a newer medication approved in 2006 for the treatment of CSA [8]. Ranolazine is recommended when therapy with beta-blockers, CCBs, and nitrates is not tolerated or contraindicated or if initial treatment is ineffective [4]. Therapy with ranolazine, alone and in combination with standard doses of beta-blockers, CCBs, and nitrates, can reduce the frequency of anginal episodes and nitroglycerin use and improve exercise duration, the time to onset of angina, ST-segment depression, and QoL in patients with CSA, with no significant effect on blood pressure or heart rate [4, 59–63]. Ranolazine is generally well tolerated. The most common adverse effects of ranolazine are constipation, nausea, headache, and dizziness [4]. Unlike beta-blockers and CCBs, ranolazine treats angina without causing significant changes to blood pressure and heart rate and it can be used in combination therapy with other antianginal agents (beta-blockers, CCBs, and nitrates) [4, 59, 60, 64]. Ranolazine is contraindicated for use in patients with corrected QT interval prolongation, clinically significant hepatic impairments, and it should not be used in combination with drugs such as ketoconazole and macrolide antibiotics that are potent inhibitors of the CYP3A4 pathway [4].

The medical indications for revascularization are the prevention of death and CV complications and the improvement of symptoms and QoL [4]. Revascularization procedures such as coronary artery bypass graft or percutaneous coronary intervention can reduce the symptoms of CSA and improve morbidity, mortality, and QoL in patients with CSA [65–67]. However, there are clinical challenges to revascularization, including restenosis/acute coronary occlusion, diffuse disease and/or poor distal target vessels, and other comorbid conditions that increase perioperative complications [57]. These challenges may preclude revascularization as a treatment option for some patients, and the results of several clinical trials have shown that angina may persist in 20% to 34% of patients even 1 year after revascularization [68–70]. Patients with recurrent angina after revascularization need treatment options beyond mechanical revascularization.

## 6. Summary 

Patients may not be receiving optimal treatment for their chronic angina and may continue to suffer from anginal episodes due in part to ineffective communication with and incomplete assessment by their healthcare provider. As a consequence, patients with chronic angina often experience impaired QoL, as they restrict their physical activities and adopt a more sedentary lifestyle in order to minimize anginal episodes. Tools designed to be used in a clinical setting that facilitate proactive and effective communication between a patient and their healthcare provider can be an asset in improving patient-provider communication. These tools can aid providers in more thoroughly assessing angina severity and the impact on a patient's QoL. In addition, these tools can help healthcare providers attain the ACCF/AHA treatment objectives of complete or nearly complete elimination of angina chest pain, with the goals of maximizing health and function and minimizing the likelihood of death in patients with CSA.

## Figures and Tables

**Table 1:** Canadian Cardiovascular Society Classification of angina according to impact on physical activity.

Level	Impact of physical activity on occurrence of angina
Class I	Ordinary physical activity, such as walking or climbing stairs, does not cause angina Angina occurs with strenuous, rapid, or prolonged exertion at work or recreation

Class II	Slight limitation of ordinary activity Angina occurs on walking or climbing stairs rapidly, walking uphill, walking or stair-climbing after meals, in cold, in wind, under emotional stress, or only during the first few hours of awakening Angina occurs on walking more than two blocks on the level and climbing more than one flight of ordinary stairs at a normal pace and in normal conditions

Class III	Marked limitations of ordinary physical activity Angina occurs on walking one to two blocks on the level and climbing one flight of stairs in normal conditions and at a normal pace

Class IV	Inability to carry out any physical activity without discomfort Anginal symptoms may be present at rest

Data from Sangareddi et al. [9].

**Table 2:** Measures of physical activity and quality of life in patients with chronic stable angina.

Variables	Control group (*n* = 441)	Stable angina group (*n* = 115)
Total LTPA (kcal/day)	212 (226)	144 (157)^a^
Mean duration LTPA (min/day)	47 (52)	34 (34)^a^
Physical Activity Scale (units)	2.3 (1.5)	1.7 (1.2)^a^
Self-perceived health (%)	80 (17)	63 (24)^a^
Physical function (%)	72 (27)	44 (21)

Data from Gardner et al. [10].

All values shown are mean (SD).

LTPA: leisure-time physical activity; SD: standard deviation.

^a^ *P* < 0.05 for patients with stable angina compared with controls after adjustment for age, race, current smoking, diabetes, hypertension, and obesity.

**Table 3:** Additional questions to assist in evaluation of patient angina status.

Additional questions to assess patient functional status^a^	
Has your activity level changed?	
Are you as active as you would like to be?	
Do you have the energy you think you should have?	
What symptoms, such as shortness of breath or fatigue, are you experiencing that limit your activity or concern you?	
What are you doing to make your angina better?	

^a^Developed by a panel of cardiac nurse practitioners and physician assistants moderated by Jeffrey Young at the CSA content development meeting, Chicago, IL, USA.

**Table 4:** Effect of antianginal treatments on myocardial oxygen supply and demand.

Drug class	O_2_ supply		O_2_ demand			Potential drug tolerance
	Coronary blood flow	Heart rate	Arterial pressure	Venous return	Myocardial contractility	
Beta-blockers	—	↓	↓	—	↓	No
DHP-CCBs	↑	↑^a^	↓	—	↓	No
Non-DHP-CCBs	↑	↓	↓	—	↓	No
Long-acting nitrates	↑	↑/—	↓	↓	—	Yes
Sodium channel inhibitor (ranolazine)	—	—	—	—	—	No
Revascularization	↑	—	—	—	↑/—	No

Adapted from Vadnais and Wenger [8], Fuster et al. [47], and Thadani and Ripley [48].

CCBs: calcium channel blockers; DHP: dihydropyridine; O_2_: oxygen.

^a^Less reflex tachycardia with amlodipine besylate.

↑: increased; ↓: decreased; —: no effect.
